# Adult exposure to passive smoking in Germany

**DOI:** 10.17886/RKI-GBE-2018-074

**Published:** 2018-06-27

**Authors:** Johannes Zeiher, Anne Starker, Thomas Lampert, Benjamin Kuntz

**Affiliations:** Robert Koch Institute, Department of Epidemiology and Health Monitoring

**Keywords:** PASSIVE SMOKING, ADULTS, EDUCATION, HEALTH MONITORING, GERMANY

## Abstract

Passive smoking is associated with the same consequences for health as smoking, albeit to a lesser extent. Various legislative measures have been put in place in Germany to lower exposure to passive smoking. According to data from GEDA 2014/2015-EHIS, 11.3% of non-smoking adults are regularly exposed to passive smoking in enclosed spaces, this is particularly the case with young adults. Non-smoking women who are regularly exposed to passive smoking usually come into contact with passive smoking when they are together with friends and acquaintances (51.2%). Non-smoking men most frequently face passive smoking in the work place (56.1%). People with a high level of education are much less frequently exposed to passive smoking than those with medium to lower levels of education. Action still needs to be taken to protect people against the dangers linked to passive smoking.

## Introduction

Tobacco smoke is one of the leading preventable causes of illness and death [1]. About one quarter of adults around the world smoke [2]. In Germany, 23.8% of adults currently smoke [3]. The consumption of tobacco products is also widespread among young people [2]. However, there is a strong tendency in Germany towards fewer numbers of young smokers: whereas more than one in five young people and one in five 11- to 17-year-olds smoked at the beginning of the millennium, this figure has now dropped to one in fourteen [4]. Smoking not only has consequences for smokers; it also comes with significant consequences for the health of people who are regularly exposed to passive smoking.

Passive smoking is defined as the involuntary inhalation of tobacco smoke from the ambient air. This smoke comes from exhaled mainstream smoke produced by active smokers, and by sidestream smoke produced when active smokers pause between smoking and leave their tobacco products to smoulder [9]. In terms of its composition, this type of smoke does not significantly differ from the smoke inhaled by active smokers, and it contains the same toxic and carcinogenic substances [9]. Numerous studies have shown that passive smoking is associated with the same effects as smoking, albeit to a lesser extent [5, 6]. Inhalation of these substances may cause acute symptoms such as headaches, dizziness, nausea or irritation of the eyes, nasal membranes and respiratory tract. The longer-term consequences that can be caused or exacerbated by regular exposure to passive smoking include various cancers and cardiovascular diseases, asthma, and chronic obstructive pulmonary disease (COPD) [9, 10]. Children who are regularly exposed to tobacco smoke are particularly at risk because they have a faster respiratory rate and lack a fully developed system of detoxification compared to adults [9]. Children who are regularly exposed to passive smoking are more frequently affected by conditions such as difficulty breathing, asthma, middle ear infections and upper respiratory tract infections [11-13]. Passive smoking is also a risk factor associated with sudden infant death syndrome [12, 14] and is associated with a higher risk of impaired perinatal development during pregnancy [15]. Around one in every hundred deaths worldwide is caused by exposure to passive smoking [7]. According to estimates, 3,330 passive smoking-related deaths occurred in Germany in 2005 [8]. More current estimates are not available.

**GEDA 2014/2015-EHIS**

**Data holder:** Robert Koch Institute

**Aims:** To provide reliable information about the population’s health status, health-related behaviour and health care in Germany, with the possibility of a European comparison

**Method:** Questionnaires completed on paper or online

**Population:** People aged 18 years and above with permanent residency in Germany

**Sampling:** Registry office sample; randomly selected individuals from 301 communities in Germany were invited to participate

**Participants:** 24,016 people (13,144 women; 10,872 men)

**Response rate:** 26.9%

**Study period:** November 2014 - July 2015

**More information in German is available at**

www.geda-studie.de

Consistent smoking bans in public places, on public transport, at work, and in the gastronomy and leisure sectors can directly reduce the burden caused by tobacco smoke [16]. Over the last 15 years, various legislative measures have been put in place in Germany [6, 9, 17]. The amendment to the Arbeitsstättenverordnung in 2002 severely restricted smoking in the workplace. In 2007, the Bundesnichtraucherschutzgesetz prohibited smoking in federal government institutions and in public railway stations. Between 2007 and 2008, the federal states issued non-smoker protection laws that banned smoking in federal government institutions, as well as in educational, sports-related, cultural and health-related facilities and in the catering trade. Although most federal states provided exemptions for the catering industry, Bavaria, Saarland and North Rhine-Westphalia have implemented complete smoking bans in the bars and restaurants under their jurisdiction.

Although non-smoker protection laws are ‘only’ aimed at reducing exposure to passive smoking, by denormalising smoking in public spaces, they can contribute to an overall decline in tobacco consumption [16]. Moreover, a low level of tobacco consumption among the population is the best protection against the dangers of tobacco smoke in ambient air. In addition to the legal protection of non-smokers, various tobacco prevention measures have been implemented since the 2000s with the aim of reducing tobacco consumption in Germany [3, 17]. These include significant increases in the tax duties placed on tobacco, advertising bans, increasing the legal age for using and buying tobacco products, stronger warnings on tobacco products and expanding setting and population-based campaigns.

## Indicator

The German Health Update (GEDA 2014/2015-EHIS) used a paper-based and online questionnaire to gather data about respondents’ exposure to passive smoking. The respondents were asked, ‘How often are you exposed to tobacco smoke in enclosed spaces?’ and were provided with the following response categories: ‘Never or almost never’, ‘Less than 1 hour a day’ or ‘1 hour a day or more’. The following sets out the results from these three categories and also subsumes the last two into a further category: ‘regular exposure to passive smoking’. The findings presented here only consider people who stated that they did not smoke. The results have been differentiated according to gender, age and educational level. The results for ‘regular exposure to passive smoking’ have been differentiated according to gender, educational level and place of exposure. Data on the place of exposure was collected using the question: ‘And where does this happen? …’ The following response categories were provided: ‘at home?’, ‘at work?’, ‘in public buildings?’, ‘in restaurants?’, ‘in pubs, cafés, bars or clubs?’, ‘with friends or acquaintances?’, and ‘or in other places?’.

The following analyses are based on data from 18,371 participating non-smokers aged 18 or older (10,262 women, 8,109 men) who provided valid information on exposure to passive smoking. Calculations were carried out using a weighting factor that corrected the sample for deviations from the population structure (on 31 December 2014) in terms of gender, age, municipality type and level of education. The municipality type reflects the degree of urbanisation in a particular area and corresponds to the way in which urbanisation is distributed throughout Germany. The International Standard Classification of Education (ISCED) was used to classify the participants’ educational and occupational qualifications [18]. A detailed description of the methodology employed for GEDA 2014/2015-EHIS can be found in Lange et al. 2017 [19] as well as in the article German Health Update: New data for Germany and Europe in issue 1/2017 of the Journal of Health Monitoring.

## Results and discussion

**Figure 1:**
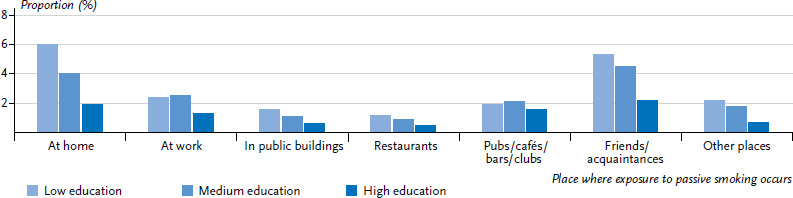
Regular exposure to passive smoking among non-smoking women according to place of exposure and educational level (n=10,262) Source: GEDA 2014/2015-EHIS

**Figure 2:**
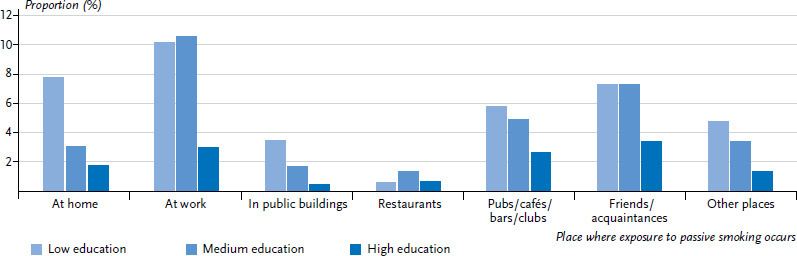
Regular exposure to passive smoking among non-smoking men according to place of exposure and educational level (n=8,109) Source: GEDA 2014/2015-EHIS

At present, 11.3% of the non-smoking adult population in Germany is exposed to passive smoking on a regular basis (Table 1 and Table 2). This includes 3.4% of non-smokers who are exposed to passive smoking for at least one hour a day and 7.9% who are exposed to passive smoking for less than one hour per day. Women are exposed less frequently to passive smoking than men (8.3% compared to 14.7%). The highest rate of exposure to passive smoking was identified among young adults aged between 18 and 29 years. Levels of exposure to passive smoking decrease with age, and this is particularly the case with people aged 65 or older. The data from GEDA 2014/2015-EHIS enables research to be undertaken regarding the places where non-smokers are exposed to passive smoking (Figure 1 and Figure 2). Women are much less likely to be exposed to passive smoking at work than men (2.2% compared to 8.2%). However, this figure underestimates the problem because it refers to the entire population [16]. Once the calculation has been limited to the population of working age (18 to 64), the proportion of non-smoking women and men exposed to passive smoking at work rises to 3.9% and 12.2%, respectively. More men than women are exposed to passive smoking in pubs, cafés, bars and clubs as well as with friends and acquaintances.

In the case of people who are regularly exposed to passive smoking, women are primarily exposed to passive smoking either when they are together with friends and acquaintances (51.2%) or at home (49.1%). In contrast, men are mainly exposed to passive smoking at work (56.1%) but also with friends and acquaintances (41.3%) (data not shown).

The data from the GEDA 2014/2015-EHIS study show clear differences according to education: both women and men with higher levels of education are exposed to passive smoking in enclosed spaces less often than those with medium to lower levels of education (Table 1 and Table 2). This difference is evident across all age groups; however, both the actual level of exposure and the differences between educational groups are markedly lower in the cases of people aged 65 years or older. The higher level of exposure to passive smoking in the lower and middle educational groups among non-smoking women is primarily due to the fact that they face an increased level of exposure at home and together with friends and acquaintances (Figure 1). Among non-smoking men, this difference can be explained by their higher level of exposure to passive smoking at work (Figure 2).

Although various studies provide an indication of temporal developments and trends in terms of passive smoking among adults, no long-term time series are currently available [6]. Data from previous GEDA survey waves demonstrate that the proportion of non-smoking adults exposed to passive smoking at least one day a week decreased from 33% to 27% between 2009 and 2012. However, since GEDA 2014/2015-EHIS employed different questions and answer categories in accordance with changes made to the European Health Interview Survey [20], these data are not comparable with those from previous GEDA surveys. Consequently, the data gathered from GEDA 2014/2015-EHIS cannot be used to study developments over time or trends in passive smoking. Data from the Epidemiological Survey on Substance Abuse also show a reduction in the level of exposure to passive smoking since the introduction of legislation protecting non-smokers. Whereas in 2006, 31% of the working population and trainees were exposed to passive smoking in the workplace, by 2009, this rate had dropped to 15%. Furthermore, the proportion of the non-smoking population exposed to passive smoking decreased from 27% to 14% during the same period; however, the proportion of non-smoking women and men exposed to tobacco smoke at home remained relatively constant (2006 11%, 2009 10%) [21, 22]. Data from two waves of the “Gesundheitsmonitor” also show a significant decrease in exposure to passive smoking between 2007 and 2014 at work, at home and during leisure time [23].

Even if it is difficult to quantify the effectiveness of individual measures, the declining trend in passive smoking, which is also evident among children and adolescents [24, 25], can be viewed as the result of improved protection for non-smokers, and stronger tobacco prevention policies in Germany. Despite these successes, there is still considerable room for improvement when it comes to non-smoker protection and prevention policy, such as in terms of nationwide advertising bans on tobacco products [26]. Major players in the field of tobacco prevention are calling for the current climate of acceptance regarding smoking bans among the population to be used to enforce uniform, nationwide non-smoking protection measures and to remove exemptions in the catering trade and the broader workplace [23, 27].

## Key statements

- A total of 8.2% of non-smoking women and 14.7% of non-smoking men are regularly exposed to passive smoking in enclosed spaces.
- In cases where non-smoking women are regularly exposed to passive smoking, this usually occurs together with friends and acquaintances (51.2%). In cases where non-smoking men are regularly exposed to passive smoking, this usually happens at work (56.1%).
- Non-smoking women and men with higher levels of education are much less frequently exposed to passive smoking than those with medium to lower levels of education.

## Figures and Tables

**Table 1:** Exposure to passive smoking among non-smoking women according to age and educational level (n=10,262) Source: GEDA 2014/2015-EHIS

Women	Never or almost never		Less than 1 hour a day		1 hour a day or more	
	%	(95% CI)	%	(95% CI)	%	(95% CI)
**Women (total)**	**91.7**	**(91.0-92.4)**	**5.2**	**(4.7-5.7)**	**3.0**	**(2.6-3.6)**
**18-29 Years**	80.2	(77.3-82.8)	13.2	(11.2-15.4)	6.6	(4.9-8.9)
Low education	70.0	(61.8-77.1)	14.9	(10.0-21.6)	15.1	(9.9-22.3)
Medium education	80.8	(77.5-83.7)	13.9	(11.4-16.8)	5.3	(3.7-7.6)
High education	90.0	(86.5-92.7)	8.8	(6.3-12.2)	1.2	(0.6-2.6)
**30-44 Years**	90.8	(89.1-92.2)	6.4	(5.2-7.9)	2.8	(2.0-4.0)
Low education	81.1	(73.0-87.2)	15.7	(10.0-23.7)	3.3	(1.3-7.9)
Medium education	90.6	(88.2-92.5)	6.1	(4.7-8.0)	3.3	(2.2-5.1)
High education	95.0	(92.6-96.6)	3.4	(2.2-5.1)	1.6	(0.7-3.8)
**45-64 Years**	92.5	(91.4-93.5)	4.3	(3.6-5.2)	3.1	(2.5-3.9)
Low education	90.4	(87.1-93.0)	5.5	(3.6-8.3)	4.0	(2.4-6.8)
Medium education	91.9	(90.3-93.3)	4.5	(3.6-5.8)	3.5	(2.7-4.7)
High education	95.9	(94.5-96.9)	2.9	(2.0-4.2)	1.2	(0.7-2.0)
**≥ 65 Years**	97.0	(96.1-97.7)	1.6	(1.1-2.2)	1.4	(1.0-2.1)
Low education	96.2	(94.4-97.5)	1.3	(0.7-2.5)	2.5	(1.6-3.9)
Medium education	97.4	(96.2-98.3)	1.8	(1.1-2.9)	0.7	(0.4-1.5)
High education	98.4	(97.0-99.2)	1.3	(0.6-2.7)	0.3	(0.1-0.9)
**Total (women and men)**	**88.7**	**(88.1-89.3)**	**7.9**	**(7.4-8.4)**	**3.4**	**(3.0-3.8)**

CI=confidence interval

**Table 2:** Exposure to passive smoking among non-smoking men according to age and educational level (n=8,109) Source: GEDA 2014/2015-EHIS

Men	Never or almost never		Less than 1 hour a day		1 hour a day or more	
	%	(95% CI)	%	(95% CI)	%	(95% CI)
**Men (total)**	**85.3**	**(84.3-86.3)**	**10.9**	**(10.0-11.8)**	**3.8**	**(3.3-4.4)**
**18-29 Years**	73.3	(69.7-76.6)	20.4	(17.4-23.6)	6.4	(4.7-8.7)
Low education	71.6	(63.1-78.9)	20.2	(14.2-27.9)	8.2	(4.8-13.8)
Medium education	71.2	(66.8-75.3)	21.7	(17.9-25.9)	7.1	(4.9-10.3)
High education	83.7	(77.2-88.6)	15.8	(10.9-22.3)	0.5	(0.1-2.6)
**30-44 Years**	83.7	(81.1-86.0)	12.0	(10.0-14.4)	4.2	(3.1-5.8)
Low education	83.9	(74.1-90.5)	12.6	(6.8-22.2)	3.5	(1.1-10.1)
Medium education	78.0	(74.2-81.4)	15.8	(12.7-19.5)	6.2	(4.3-8.9)
High education	92.3	(89.7-94.3)	6.2	(4.4-8.7)	1.5	(0.8-2.8)
**45-64 Years**	85.8	(84.2-87.2)	10.8	(9.5-12.2)	3.4	(2.7-4.4)
Low education	76.7	(70.1-82.3)	15.5	(11.2-21.2)	7.8	(4.8-12.3)
Medium education	82.8	(80.4-85.0)	13.5	(11.6-15.7)	3.7	(2.7-5.1)
High education	92.9	(91.1-94.3)	5.2	(4.0-6.7)	1.9	(1.2-3.1)
**≥ 65 Years**	93.0	(91.8-94.1)	4.5	(3.7-5.6)	2.4	(1.8-3.2)
Low education	92.5	(89.5-94.8)	5.1	(3.4-7.7)	2.3	(1.1-4.7)
Medium education	93.1	(91.2-94.6)	4.5	(3.3-6.1)	2.5	(1.6-3.7)
High education	93.2	(91.1-94.8)	4.4	(3.2-6.2)	2.4	(1.6-3.6)
**Total (women and men)**	**88.7**	**(88.1-89.3)**	**7.9**	**(7.4-8.4)**	**3.4**	**(3.0-3.8)**

CI=confidence interval

## References

[1] Jha P Peto R (2014) Global Effects of Smoking, of Quitting, and of Taxing Tobacco. New England Journal of Medicine 370(1):60-68 10.1056/NEJMra1308383 24382066

[2] Reitsma MB Fullman N Ng M (2017) Smoking prevalence and attributable disease burden in 195 countries and territories, 1990-2015: a systematic analysis from the Global Burden of Disease Study 2015. The Lancet 389(10082):1885-1906 10.1016/S0140-6736(17)30819-X PMC5439023 28390697

[3] Zeiher J Kuntz B Lange C (2017) Smoking among adults in Germany. Journal of Health Monitoring 2(2):57-63. https://edoc.rki.de/handle/176904/2664 (As at 29.05.2018) 10.17886/RKI-GBE-2017-043 PMC10161277 37152097

[4] Zeiher J Starker A Kuntz B (2018) Smoking behaviour among children and adolescents in Germany. Results of the cross-sectional KiGGS Wave 2 study and trends. Journal of Health Monitoring 3(1):38-44. https://edoc.rki.de/handle/176904/5630 (As at 29.05.2018) 10.17886/RKI-GBE-2018-025 PMC8848845 35586175

[5] Fischer F Kraemer A (2015) Meta-analysis of the association between second-hand smoke exposure and ischaemic heart diseases, COPD and stroke. BMC Public Health 15:1202 26627181 10.1186/s12889-015-2489-4 PMC4667413

[6] Kuntz B Zeiher J Starker A (2017) Passivrauchbelastung der Bevölkerung in Deutschland: 10 Jahre Bundesnichtraucher-schutzgesetz. Epidemiologisches Bulletin 2017(33):325-329

[7] Öberg M Jaakkola MS Woodward A (2011) Worldwide burden of disease from exposure to second-hand smoke: a retrospective analysis of data from 192 countries. The Lancet 377(9760):139-146 10.1016/S0140-6736(10)61388-8 21112082

[8] Keil U Prugger C Heidrich J (2016) Passivrauchen Public Health Forum, Vol 24, P. 84

[9] Deutsches Krebsforschungszentrum (2015) Tabakatlas Deutschland 2015. Pabst, Heidelberg

[10] U.S. Department of Health and Human Services (2014) The health consequences of smoking - 50 years of progress: a report of the Surgeon General. U.S. Department of Health and Human Services, Centers for Disease Control and Prevention, National Center for Chronic Disease Prevention and Health Promotion, Office on Smoking and Health, Atlanta, Georgia, USA

[11] Burke H Leonardi-Bee J Hashim A (2012) Prenatal and Passive Smoke Exposure and Incidence of Asthma and Wheeze: Systematic Review and Meta-analysis. Pediatrics 129(4):735-744 22430451 10.1542/peds.2011-2196

[12] Polanska K Hanke W Ronchetti R (2006) Environmental tobacco smoke exposure and children’s health. Acta Paediatr Suppl 95:86-92 17000575 10.1080/08035320600886562

[13] Jones LL Hassanien A Cook DG (2012) Parental smoking and the risk of middle ear disease in children: A systematic review and meta-analysis. Archives of Pediatrics & Adolescent Medicine 166(1):18-27 21893640 10.1001/archpediatrics.2011.158

[14] Treyster Z Gitterman B (2011) Second hand smoke exposure in children: environmental factors, physiological effects, and interventions within pediatrics. Rev Environ Health 26(3): 187-195 22206195 10.1515/reveh.2011.026

[15] Salmasi G Grady R Jones J (2010) Environmental tobacco smoke exposure and perinatal outcomes: a systematic review and meta-analyses. Acta Obstet Gynecol Scand 89(4):423-441 20085532 10.3109/00016340903505748

[16] Lampert T List S (2010) Health Risks of Environmental Tobacco Smoke Exposure. GBE kompakt 3(1). Robert Koch Institute, Berlin. https://edoc.rki.de/handle/176904/3102 (As at 29.05.2018)

[17] Kuntz B Zeiher J Starker A (2018) Tabak - Zahlen und Fakten zum Konsum. In: Deutsche Hauptstelle für Suchtfragen e.V. (Ed) DHS Jahrbuch Sucht 2018. Pabst, Lengerich, P. 50-84

[18] Statistical Office of the European Union (Eurostat) (2016) International Standard Classification of Education (ISCED). http://ec.europa.eu/eurostat/statistics-explained/index.php?title=Glossary:International_standard_classification_of_education_(ISCED) (As at 20.02.2018)

[19] Lange C Finger JD Allen J (2017) Implementation of the European health interview survey (EHIS) into the German health update (GEDA). Arch Public Health 75:40 28936356 10.1186/s13690-017-0208-6 PMC5603169

[20] Robert Koch-Institut (2017) Fragebogen zur Studie „Gesundheit in Deutschland aktuell“: GEDA 2014/2015-EHIS. Journal of Health Monitoring 2(1):105-135. https://edoc.rki.de/handle/176904/2587 (As at 29.05.2018)

[21] Kraus L Pabst A Piontek D (2010) Kurzbericht Epidemiologischer Suchtsurvey 2009. Tabellenband: Prävalenz von Tabakkonsum, Nikotinabhängigkeit und Passivrauchen nach Geschlecht und Alter im Jahr 2009. IFT Institut für Therapieforschung, München. https://esa-survey.de/fileadmin/user_upload/Literatur/Berichte/ESA_2009_Tabak-Kurzbericht.pdf (As at 20.02.2018)

[22] Baumeister SE Kraus L Stonner T (2008) Tabakkonsum, Nikotinabhängigkeit und Trends. Ergebnisse des Epidemiologischen Suchtsurveys 2006. SUCHT 54(7):26-35

[23] Schaller K Braun S Pötschke-Langer M (2014) Erfolgsgeschichte Nichtraucherschutz in Deutschland: Steigende Unterstützung in der Bevölkerung für gesetzliche Maßnahmen. Gesundheitsmonitor 4/2014. https://www.bertelsmann-stiftung.de/fileadmin/files/Projekte/17_Gesundheitsmonitor/Gesundheitsmonitor_NL_4_2014.pdf (As at 20.02.2018)

[24] Kuntz B Lampert T (2016) Social disparities in parental smoking and young children’s exposure to secondhand smoke at home: a time-trend analysis of repeated cross-sectional data from the German KiGGS study between 2003-2006 and 2009-2012. BMC Public Health 16:485 27277721 10.1186/s12889-016-3175-x PMC4898452

[25] Kuntz B Lampert T (2016) Smoking and passive smoke exposure among adolescents in Germany. Prevalence, trends over time, and differences between social groups. Deutsches Ärzteblatt International 113(3):23-30 26857509 10.3238/arztebl.2016.0023 PMC4748148

[26] Joossens L Raw M (2017) The tobacco control scale 2016 in Europe. Association of European Cancer Leagues (ECL), Brussels

[27] Schaller K Effertz T Gerlach S (2016) Prävention nichtübertragbarer Krankheiten – eine gesamtgesellschaftliche Aufgabe. Grundsatzpapier der Deutschen Allianz Nichtübertragbare Krankheiten (DANK). DANK, Berlin. www.dank-allianz.de/files/content/dokumente/DANK-Grundsatzpapier_ES.pdf (As at 20.02.2018)

